# Prevalence of Mastitis in Cows and Raw Milk Microbial Contamination in Selected Districts, Tanzania

**DOI:** 10.1002/vms3.71138

**Published:** 2026-08-05

**Authors:** C. Faustine Ndyamukama, G. Msalya, Hezron Emmanuel Nonga

**Affiliations:** ^1^ Department of Animal Aquaculture and Range Sciences, Sokoine University of Agriculture Morogoro Tanzania; ^2^ Tanzania Dairy Board Dodoma Tanzania; ^3^ Department of Veterinary Medicine and Public Health College of Veterinary Medicine and Biomedical Sciences, Sokoine University of Agriculture Morogoro Tanzania

**Keywords:** antimicrobial resistance, mastitis, milk, milk contamination, udder health

## Abstract

**Background:**

Milk production from cows in Tanzania may not have reached optimum levels due to several constraints, including diseases such as mastitis.

**Objectives:**

A cross‐sectional study was conducted in the Kilosa, Mvomero, Lushoto and Handeni districts to establish the prevalence of mastitis, milk bacteria contamination and antimicrobial resistance in lactating cows.

**Methods:**

A total of 78 cows were examined for mastitis using udder palpation and California Mastitis Tests. Subsequently, milk samples were collected for bacteriological culture and antimicrobial susceptibility tests using standard protocols.

**Results:**

The prevalence of mastitis in cows was 75.6%, dominated by subclinical mastitis, which accounted for 62.8% (*p* = 0.02732). Dairy cows were more affected by mastitis (82.1%) than local breeds (72%). Up to 78% of mastitis milk samples had bacterial contamination. A total of 137 bacterial isolates detected were categorised as non‐mastitis (27.0%), subclinical (56.2%) and clinical (16.8%). The seven bacterial species identified were *Staphylococcus aureus* (20.4%), *Staphylococcus epidermidis* (19.7%), *Listeria monocytogenes* (18.2%), *Listeria innocua* (17.5%), *Listeria ivanovii* (9.5%), *Streptococcus agalactiae* (7.2%) and *Escherichia coli* (7.3%). Most of the milk samples (78.5%) containing bacterial isolates exhibited mixed infections. Up to 56.9% of milk samples from Tanzania Short Horned Zebu (TSHZ) contained mixed bacterial species. Cows from the Kilosa District had more isolates (33.6%) than the rest. More than 70% of the bacterial isolates were resistant to tetracycline, nalidixic acid and amoxycillin. *S. aureus*, *S. epidermidis*, *E. coli* and *L. monocytogenes* showed the highest resistance rates against amoxycillin/clavulanic acid, ampicillin and amoxycillin.

**Conclusions:**

A high prevalence of mastitis implies losses to farmers due to poor production and threatens public health from milk‐borne diseases. This calls for urgent intervention measures to control mastitis, combat antimicrobial resistance and safeguard public health.

## Graphical abstract

Cow milk produced in Tanzania suffers from poor quality due to bacterial contamination from mastitis udder predisposed by poor livestock husbandry and milk hygienic practices. The prevalence of mastitis was 75.6%, with a milk bacterial contamination rate of 78% and resistance to multiple antimicrobials. To safeguard the dairy industry and public health, farmers and other stakeholders are obliged to address the problem of mastitis in cows.

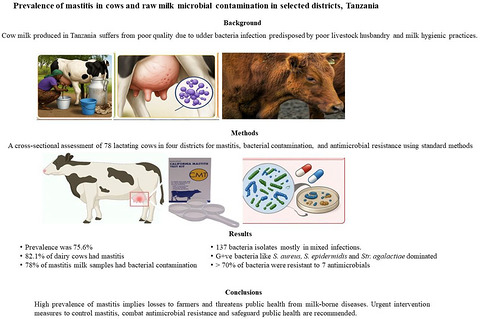

## 1 Introduction

Tanzania has a large livestock resource, including 40 million cattle, of which dairy cattle are estimated at 3%–4% (Mlozi et al. 2015; Mramba and Mohamed 2024; Ministry of Livestock and Fisheries [MLF] 2025). The annual growth rate of the livestock sector is 3.4%, and its contribution to the gross domestic product (GDP) is 7.4% (MLF 2025; TLMP 2017). Annual milk production is still relatively low and estimated to be 4.1 billion litres and mostly comes from indigenous cattle (MLF 2025). This low milk production may be caused by reasons including poor genetic potential of livestock, limited availability of good quality feeds, livestock diseases and generally poor management (Njombe et al. 2011; Maleko et al. 2018). Furthermore, the post‐harvest losses of milk are between 16% and 25% and are caused by different factors, including poor livestock husbandry practices, poor milk hygienic practices along the value chain, lack of cold chain and market problems (Lugamara 2024). Tick‐borne diseases (TBDs), in particular East Coast fever, babesiosis, anaplasmosis and ehrlichiosis, are of great importance to livestock productivity in Tanzania (Kivaria et al. 2007; Nonga et al. 2012; Ozbey et al. 2022). Mastitis in clinical or subclinical forms is another important disease that compromises the dairy sector worldwide and is more prevalent in dairy cattle (Mramba and Mohamed 2024). Subclinical mastitis is the most prevalent in cattle and causes a 70% reduction in milk yield and a significant increase in somatic cell count (SCC) (Mbeho 2012; Ozbey et al. 2022). Clinical mastitis is associated with an inflamed and discoloured udder and changes in milk due to the presence of blood, pus, flakes and clots (Zigo et al. 2019; Ozbey et al. 2022; Mramba and Mohamed 2024). The economic consequences of mastitis include reduced milk production, poor quality milk, increased culling rate and increased cost of veterinary services and medicine (Rahman et al. 2009).

Clinical or subclinical mastitis in cattle is caused by microorganisms, mostly bacteria, including *Staphylococcus* spp., *Streptococcus* spp., *Escherichia coli*, *Actinobacter pyogenes*, *Pseudomonas aeruginosa*, *Proteus* spp. and many others (Abdel‐Rady and Sayed 2009; Hosseinzadeh and Saei 2014; Ashraf and Imran 2020). Bacteria are always shed in milk, where they pose a potential threat in causing milk‐borne diseases to humans. Occasionally, mastitis is also associated with milk‐borne zoonotic infections such as tuberculosis and brucellosis (Ashraf and Imran 2020). Factors driving mastitis occurrence include poor udder and milking hygiene and environmental hygiene of lactating cows (Mbindyo et al. 2020; Khasapane et al. 2023).

Nonetheless, microbial contamination of milk may originate from animals themselves or along the milk value chain (Abdel‐Rady and Sayed 2009; Sohrabi et al. 2013). Water used in sanitary activities along the milk production chain is among the important sources of milk contamination. In addition, recontamination may also occur after milk processing due to unhygienic conditions, improper handling and storage of milk during consumption (Ngasala et al. 2015). Therefore, routine monitoring of the microbiological status of milk at all stages of the value chain is essential to safeguard the health of the public and minimise losses due to milk condemnations. The prevailing disease burden in smallholder dairy farms, especially mastitis associated with improper livestock husbandry practices, fuels frequent use of antimicrobials as prophylaxis and treatment options (Njombe et al. 2011; Sohrabi et al. 2013; Mlozi et al. 2015; Ngasala et al. 2015; Ashraf and Imran 2020). This indiscriminate use of antimicrobials has resulted in the development of antimicrobial resistance (AMR) in animals and humans (Mlozi et al. 2015; Ashraf and Imran 2020; Sohrabi et al. 2013; Ngasala et al. 2015).

Cow milk produced in Tanzania suffers from poor quality mainly caused by microbes, drug residues or physical contamination that pose public health threats and deter the export of milk and milk products (Njombe et al. 2011; Ngasala et al. 2015; Gwandu et al. 2018; Ukita et al. 2021; Mengele et al. 2023). Drug residues, particularly those arising from the indiscriminate use of antimicrobials, contribute to the development of AMR, an emerging global threat to both animal and public health. The most frequently used antimicrobials in cattle production are sulphonamides, tetracyclines and fluoroquinolones (Kimambo et al. 2026; Mhozya and Nonga 2026; Mashauri et al. 2025). Oxytetracycline is the single most dominant antibiotic, accounting for over two‐thirds of all veterinary antibiotics consumed in the country, and this may be the reason for most bacteria developing resistance against the medicine (Sangeda et al. 2021). Therefore, this study was conducted to estimate the prevalence of mastitis, assess bacterial milk contamination and test for bacterial AMR in selected districts in Tanzania.

## 2 Materials and Methods

### 2.1 Study Areas, Population and Design

The selection of study districts was based on the availability of livestock keepers in extensive and semi/intensive production systems that involve agro‐pastoralists and smallholder dairy farmers who supply milk to the local community and nearby milk processing plants. The four districts purposely selected were Kilosa and Mvomero in the Morogoro region and Lushoto and Handeni in the Tanga region. Furthermore, the selected districts represent the other districts in Tanzania with smallholder dairy farmers and agro‐pastoralists raising cattle under similar farming systems.

Four villages from each of the four districts were purposely selected by the Safe Food, Fair Food (SFFF II) project based on criteria of the number of livestock keepers and accessibility. With the help of Ward Livestock Field Officers (LFO), households with lactating cows in the selected villages were identified and a list was prepared in each village that was used as the sampling frame. Smallholder farmers in the specific village were obtained by a random selection procedure from the sampling frame. The project plan was to visit 25 households with lactating cows in each of the four project districts. Household inclusion criteria comprised smallholder farmers with at least one lactating cow during the study period, willingness to participate and availability of milk at the time of data collection.

### 2.2 Description of Study Districts

**FIGURE 1:**
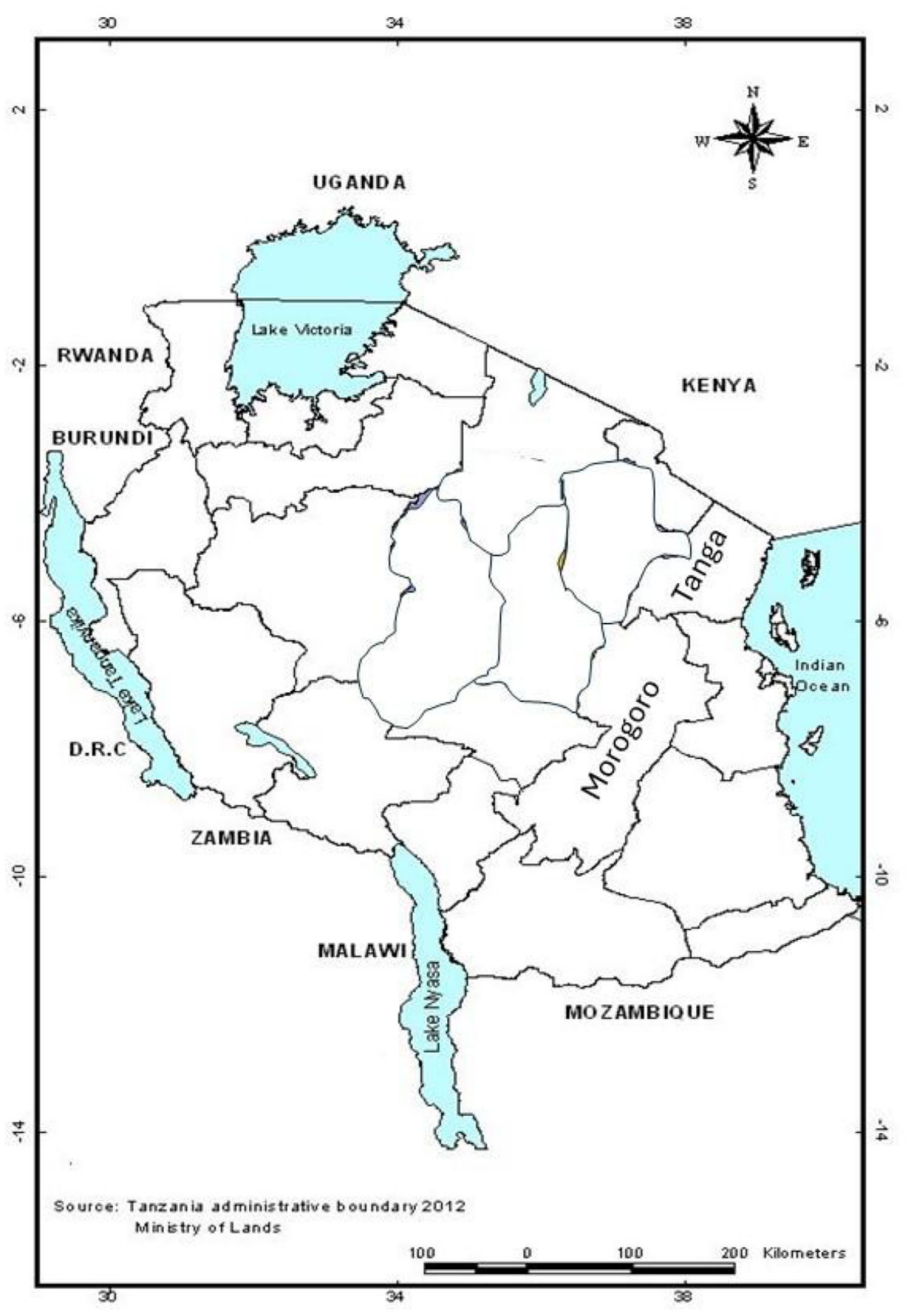
A map of Tanzania showing the locations of the Morogoro and Tanga regions, where the Kilosa and Mvomero districts and the Handeni and Lushoto districts are located, respectively.

Kilosa and Mvomero are located in the Morogoro region (Figure 1). According to the 2022 National census, the Kilosa District has a human population of 617,032, while the Mvomero District has a population of 421,741 (Population and Housing Census Tanzania 2022). The annual average rainfall in the districts ranges between 500 and 1800 mm, and the ambient temperature ranges from 27°C to 31°C. There is a bimodal rain pattern, with approximately 83% of the rainfall between late February and the end of May and short rains between November and January. Handeni and Lushoto districts are located in the Tanga region (Figure 1). Lushoto is a mountainous area 1200–1900 m above sea level, with a mean annual rainfall of 800–1400 mm (ILRI 2007). It has a human population of 492,441. Handeni District, on the other hand, is in the lowlands (500–900 m above sea level) and is much drier, with a human population of 384,353. Generally, the Handeni and Lushoto districts experience a bimodal rainfall pattern, with the long rainy season occurring from March to May and the short rainy season from October to December, while dry seasons extend from June to September and January to February. Annual rainfall ranges between 800 and 1400 mm, with temperatures varying from 18°C to 32°C (Weather Atlas 2025).

### 2.3 Study Animals, Population and Sample Size

The cattle involved in the study were lactating cows from agro‐pastoralists and smallholder dairy farmers. Agro‐pastoralists mostly kept local cattle, mostly Tanzania Short Horned Zebu (TSHZ), while smallholder dairy farmers had crosses of Friesian, Ayrshire and Jersey cattle. The TSHZs were raised under an extensive grazing system on communal grazing lands. All smallholder livestock farmers practice hand milking within the cattle houses. Smallholder dairy farmers raised their cattle under semi or intensive systems. During visits to farmers to identify households with lactating cows, it was observed that most dairy cows were housed on concrete floors without bedding, whereas all TSHZ cattle were kept on earthen floors with accumulated manure. Cattle breeding is consistently carried out through natural mating with bulls.

A formula by Thrusfield (2005), $n=Z^{2}P{\left(1-P\right)}^{2}/d^{2}$, was used to calculate the sample size of households for this study. Here, n is the required sample size, Z is the standard normal deviation for the desired confidence level (e.g., 1.96 for 95% confidence), P is the expected prevalence, which was estimated at 12.8% (Karimuribo et al. 2006) and d is the desired absolute precision (margin of error, e.g., 0.05 for ±5%). Calculations:

- Compute variance term: P(1−P)=0.128×(1−0.128)=0.128×0.872=0.1117
- Multiply by $Z^{2}$: $Z^{2}\times P\left(1-P\right)=1.96^{2}\times 0.1117=3.8416\times 0.1117=0.4289$
- Divide by $d^{2}$: $n=\frac{0.4289}{0.05^{2}}=\frac{0.4289}{0.0025}=171.6$

The calculated sample size was 171 cows.

Although the calculated sample size was 171 households (equivalent to 171 lactating cows), the Safe Food, Fair Food (SFFF II) project planned to screen only 100 cows due to seasonal non‐lactation, limited availability of eligible animals, budgetary constraints and the complex geographical distribution across the four districts. During study implementation, only 78 lactating cows from 78 households were screened, as most households in the study villages had non‐lactating cows during the dry season.

In each study household, a single lactating cow was randomly selected for mastitis screening. Study cattle were randomly selected from household herds, ensuring that each eligible lactating cow had an equal chance of inclusion. Randomisation was achieved by assigning numbers to all lactating cows within a herd and then using a random number generator to select one for mastitis screening. The selected cows were examined for clinical and subclinical mastitis.

### 2.4 Examination of Cows for Mastitis

All the selected cows were examined for clinical and subclinical mastitis. Examination of clinical mastitis involved visual and palpation of the udder for abnormalities such as swelling, hardness, heat, redness, or pain and lesions on the teats. Stripping teats for a few squirts of milk into a strip cup was enhanced to check for abnormalities such as watery appearance, clotted/blood stained milk, changes in colour, clots and flakes. A cow showing any reaction to these signs and tests was regarded as a clinical mastitis case.

Screening for subclinical mastitis was performed using the California Mastitis Test (CMT). Briefly, milk was drawn from each quarter into the corresponding CMT white plastic paddle, approximately 2 mL in each receptacle (Table 1). The CMT reagent (Shalma test reagent R) was squirted without frothing from a CMT reagent dispenser until an amount equal to the volume of milk in each of the test cups was attained. Mixing was performed by gentle circular motion of the paddle in a horizontal plane. The reaction developed its peak colour within 10 s. Each sample test was graded, interpreted and scored in four categories as in Table 1. A cow that reacted to these tests in either quarter was classified as having subclinical mastitis.

**TABLE 1:** California Mastitis Test scores and equivalent somatic cell counts.

CMT test score	Reaction observed	Equivalent milk, SCC/mL	Health quarter
Negative	Homogeneous mixture, no thickening	0–200,000, 0%–25% neutrophils	Health quarter
Trace	Slight (a faint cloud‐like) thickening that disappear approximately 10 min	150,000–500,000, 30%–40% neutrophils	Possible infection if all quarters read trace, there is infection. If one or two quarters read trace, infection is possible
1	Distinct slime formation occurs immediately after mixing, but there is no tendency to form gel; this slime does not disappear over time	400,000–500,000, 30%–60% neutrophils	Subclinical mastitis
2	Distinct slime formation occurs immediately after mixing with a slight gel formation	500,000–800,000, 60%–70% neutrophils	Subclinical mastitis
3	Distinct slime formation occurs immediately after mixing, the distinct gel is formed and the surface of the mixture becomes elevated like fried egg	> 800,000, 70%–80% neutrophils	Subclinical mastitis

Abbreviations: CMT, California Mastitis Test; SCC, somatic cell count.

*Source*: Kivaria et al. (2007).

### 2.5 Collection of Milk Samples

After examination for mastitis, all 78 study cows comprising dairy (*n* = 28) and TSHZ cows (*n* = 50) were sampled. Up to 45 mL of milk samples were collected aseptically from each quarter and mixed in one sterile screw‐capped Falcon tube. Cows that were undergoing treatment or had received antimicrobial therapy within the preceding 2–4 days were excluded from sampling. All collected samples were coded with numbers for identification and packed in a cool box with ice packs. Thereafter, the samples were transported to Sokoine University of Agriculture (SUA) laboratories and stored at −20°C until analysis.

### 2.6 Laboratory Analysis of Milk Samples

Laboratory analysis of milk samples was performed in the Microbiology Laboratory of the College of Veterinary Medicine and Biomedical Sciences, Sokoine University of Agriculture. The target bacteria were *E. coli*, *Staphylococcus aureus*, *Streptococcus* spp. and *Listeria* spp.

#### 2.6.1 Preparation of Initial Suspension and Positive Control

Milk samples were thawed at room temperature, and 10 mL was transferred into a sterile conical flask containing 90 mL of peptone water (PW) and mixed well. Furthermore, 1 mL of the initial inoculum was diluted by adding it into a test tube with 9 mL of PW and further diluted to 10^−5^ before microbiological tests of *E. coli*, *S. aureus* and *Streptococcus* spp. For *Listeria* spp., the thawed milk samples were enriched by adding 10 mL into a sterile beaker containing 90 mL of Half Fraser broth and incubated at 30°C for 24 h. Subculture enrichment was performed by adding 0.1 mL of pre‐enriched sample into a sterile test tube containing 10 mL of Fraser broth and incubating at 37°C for 48 h.

The positive control sample consisted of 500 mL collected from Magadu Farm at SUA and was boiled for 10 min, cooled and placed in a sterile bottle. Three aliquots of boiled milk of 30 mL each were prepared in test tubes and inoculated with reference strains of *E. coli* ATCC 2262‐79 (DEC9B), *S. aureus* NCTC 6571/ATCC 9144, *Streptococcus* sp. 15185 and *Listeria monocytogenes* NCTC 13372/ATCC 7644 (TCS Biosciences). The inocula were diluted as for the samples for the test bacteria above.

#### 2.6.2 Identification of the Test Bacteria in Milk

Detection of *E. coli* was performed in accordance with ISO 21528‐2:2004 protocols (ISO 2004). Paired sterile Petri dishes were supplemented with 1 mL of the test sample and shaken to spread and 15 mL of Violet Red Bile Glucose Agar (VRBG) was added and allowed to solidify. The inoculated plates were incubated at 37°C for 24 h. Typical pink to red, with or without precipitation haloes or colourless mucoid colonies, with a diameter of 0.5 mm or more, were subcultured onto nutrient agar (NA) and MacConkey agar (MCA) plates and incubated under similar conditions for biochemical tests. Gram stain was performed, followed by biochemical tests such as IMViC tests (indole, methyl Red, Voges–Proskauer, citrate), catalase, oxidase and glucose tests.

Detection and identification of *S. aureus* in milk samples was performed by using ISO 6888‐1:1999 protocols (ISO 1999). It involved inoculation of 1 mL of the test sample on Baird‐Parker (BP) agar and incubation as above. Typical colonies were black or grey, shiny, convex in shape and surrounded by a clear zone with an opalescent ring. The bacterial colonies were Gram‐stained and examined under a light microscope. Confirmation of *S. aureus* was performed by subculture on brain heart infusion (BHI) broth followed by a coagulase test.

Detection of *Streptococcus* spp. followed the same procedures as for *Staphylococcus* spp. The latex agglutination test was performed for the quick identification of *Streptococcus* spp. colonies, whereby a positive result involved the appearance of strong agglutination of the blue latex particles. Furthermore, differentiation between *Staphylococcus* and *Streptococcus* genera was carried out using the catalase test.

Identification of *L. monocytogenes* in milk samples was performed in accordance with ISO 11290‐1:1996 protocols (ISO 1996). Fraser broth cultures were inoculated onto Oxford agar and Colorex *Listeria* agar and incubated at 37°C for 24 and 48 h. Colonies presumed to be *Listeria* spp. on Oxford agar appeared small, brown‒green to dark‐brown, surrounded by black halos. Specifically, for *L. monocytogenes* and *Listeria ivanovii* on Colorex *Listeria* agar, the colonies appeared blue green in colour with well‐defined edges surrounded by opaque white halos. A *Listeria* Test kit (Oxoid Ltd., Basingstoke, Hampshire, England, Ref DR1126A, Lot 1239689) was used for the presumptive identification of *Listeria*. Confirmation of presumed *Listeria* colonies was performed by a series of biochemical tests that included haemolysis, CAMP, oxidase and Listeria latex agglutination tests (Jamali et al. 2013; Khan et al. 2013).

#### 2.6.3 Determination of Antimicrobial Susceptibility of Isolated Milk‐Borne Bacteria

All isolated milk‐borne bacteria, namely, *S. aureus*, *Staphylococcus epidermidis*, *Streptococcus agalactiae*, *E. coli*, *L. monocytogenes*, *L. ivanovii* and *Listeria innocua*, were tested for antimicrobial susceptibility to a panel of seven commonly used antimicrobials in veterinary practices. The discs used included Oxoid Ltd. (Basingstoke, Hampshire, England), tetracycline (TE: 30 µg), gentamycin (CN: 10 µg), ciprofloxacin (CIP: 5 µg), amoxycillin/clavulanic acid (AMC: 30 µg), nalidixic acid (NA: 30 µg), ampicillin (AMP: 10 µg) and amoxycillin (AML: 10 µg).

The Kirby‐Bauer disk diffusion method was used as described by the Clinical Laboratory Standards Institute (CLSI 2007) guidelines. Briefly, the isolates were subcultured onto NA and incubated at 37°C for 24 h, and then two colonies were picked using a sterile wire loop, introduced into 5 mL of normal sterile saline in universal bottles and mixed. The turbidity of the mixture was then compared with a 0.5 McFarland standard using a Vitek colourimeter (Lenexa, KS, USA). Then, 200 µL of the suspension was dispensed onto Mueller–Hinton agar plates and spread by using a sterile glass spreader for confluent growth as described by Luangtongkum et al. (2007). After drying the plates with inocula for a few minutes, antibiotic discs were placed over inoculated plates using a disc dispenser (Oxoid Ltd., Basingstoke, Hampshire, England) and sterile forceps. Thereafter, the plates were inverted and incubated under aerobic conditions at 37°C for 24 h. After the incubation period, the plates were examined for zones of inhibition and measured in millimetres (mm) using a metal calliper. The results were recorded as resistant or sensitive to the tested drugs according to the CLSI (2007) recommendations. Standard reference strains of *S. aureus* NCTC 6571/ATCC 9144, *Streptococcus* sp. 15185, *E. coli* (ATCC 25922) and *L. monocytogenes* NCTC 13372/ATCC 7644 (TCS Biosciences) were used as quality control organisms in antimicrobial susceptibility determination.

## 3 Data Management and Analysis

Collected data were verified, cleaned and entered into Microsoft Excel. Descriptive statistics (percentages and frequencies) were used to describe and summarise the data. The proportions considered included district of origin of cows, breed, nature of mastitis (clinical or subclinical), bacterial species and their sources. The data were analysed using Epi‐Info version 7.2.6.0 (CDC, USA). A binary logistic regression analysis was conducted to determine the predictors of the presence of mastitis‐positive cases in lactating cows. The predictor variables included district of origin, breed and grazing system. Chi‐square (*χ* ^2^) and confidence intervals were used to compare proportions of categorical variables such as risk factors for mastitis; a probability of *p* < 0.05 was statistically significant.

## 4 Results

### 4.1 General Results

A total of 78 cattle were examined for mastitis, and milk samples were collected for laboratory examination (Table 2). The majority of cows were from Handeni and Kilosa, dominated by local breeds (TSHZ), which were extensively grazed.

**TABLE 2:** Basic parameters of cows in the study districts (*n* = 78).

Parameter	Category	Number of lactating examined	Percentage
District	Handeni	25	32.1
	Kilosa	25	32.1
	Lushoto	10	12.8
	Mvomero	18	23.0
Breed	Dairy	28	35.9
	TSHZ	50	64.1
Grazing system	Extensive	53	68.0
	Intensive/semi‐intensive	25	32.0

### 4.2 Prevalence and Factors for Occurrence of Mastitis in Cows

The status of mastitis in individual cows in the four districts studied is shown in Table 3. Of all lactating cows examined, 59 (75.6%) had mastitis. Subclinical mastitis accounted for 62.8% of the cows examined, and the difference was statistically significant (*p* = 0.02732). Dairy cows were more affected by mastitis (82.1%, 23/28) than by TSHZ (72%, 36/50), but the difference was not statistically significant (*p* = 0.31679). More mastitis cases were encountered in Handeni District (28.2%, 22/78). Clinical mastitis was common in dairy cows (30.4%), while subclinical mastitis was common in TSHZ (91.6%).

**TABLE 3:** Status of mastitis in milk from individual cows.

Parameter	Category	Number (%) of mastitis in study districts				Total	*χ* ^2^	*p*
		Handeni	Kilosa	Lushoto	Mvomero			
Type of mastitis	Subclinical	20 (25.6)	14 (17.9)	7 (8.9)	8 (10.3)	49 (62.8)	4.8700	0.0273
	Clinical	2 (2.6)	1(1.3)	0 (0.0)	7 (8.9)	10 (12.9)		
Mastitis in cow breeds	TSHZ (*n* = 50)	20 (40.0)	15 (30.0)	0 (0.0)	1 (2.0)	36 (72.0)	1.0022	0.3168
	Dairy (*n* = 28)	2 (7.1)	0 (0.0)	7 (27)	14 (50.0)	23 (82.1)		
Mastitis per type of cow grazing system	Extensive	20 (25.6)	15 (19.2)	0 (0.0)	3 (3.9)	38 (48.7)	1.3952	0.2375
	Intensive/semi‐intensive	2 (2.6)	0 (0.0)	7 (9.0)	12 (15.4)	21 (26.9)		
Mastitis in dairy cows	Subclinical	2 (2.6)	0 (0.0)	7 (8.9)	7 (8.9)	16 (57.1)		
	Clinical	0 (0.0)	0 (0.0)	0 (0.0)	7 (8.9)	7 (25.0)		
Mastitis in TSHZ cows	Subclinical	18 (23.1)	14 (17.9)	0 (0.0)	1 (1.3)	33 (66.0)		
	Clinical	2 (2.6)	1 (1.3)	0 (0.0)	0 (0.0)	3 (6.0)		

### 4.3 Binary Logistic Regression Analysis of Risk Factors for Mastitis

A binary logistic regression analysis was conducted to determine the predictors of the presence of mastitis‐positive cases (Table 4). The final model was not statistically significant according to the omnibus test of model coefficients (*χ* ^2^ (5) = 8.50, *p* = 0.131). The model explained 15.4% of the variance in mastitis status (Nagelkerke *R* ^2^). Despite the lack of overall significance, the model correctly classified 76.9% of the cases. The Hosmer and Lemeshow goodness‐of‐fit test was non‐significant (*χ* ^2^ (4) = 1.23, *p* = 0.873), indicating that the model's predictions did not significantly differ from the observed data. None of the individual predictors were statistically significant factors for mastitis.

**TABLE 4:** Logistic regression predicting mastitis.

						95% CI	
Parameter	Category	*B*	SE	*p*	OR	Lower	Upper
Geographical location	Mvomero (ref)						
	Handeni	1.97	1.49	0.186	7.20	0.39	133.88
	Kilosa	0.47	1.43	0.743	1.60	0.19	26.53
	Lushoto	−1.17	1.04	0.260	0.31	0.04	2.38
Breed	Zebu (ref)						
	Dairy	1.19	1.91	0.532	3.30	0.08	139.39
Grazing	Extensive (ref)						
	Intensive	1.01	1.40	0.472	2.74	0.18	42.64
Constant		1.13	1.134	0.399	3.09		
						

*Note: N* = 78.

Abbreviations: *B*, unstandardised regression coefficient; CI, confidence interval; OR, odds ratio; SE, standard error.

### 4.4 Bacteria Isolates in Cow Milk

The bacterial isolates in milk from mastitis and non‐mastitis dairy and TSHZ cows in the study districts are shown in Tables 5 and 6. It was established that 64 (82.1%) of the milk samples had bacterial contamination. A higher bacterial isolation rate (59%; *n* = 46) was established in milk samples from mastitis cows than in those from normal cows (23.1; *n* = 18). Interestingly, 13 milk samples that were positive for mastitis had no bacterial contamination. The bacterial isolation rate in milk from cows with mastitis was 78% (46/59).

**TABLE 5:** Bacteria isolates in mastitis and non‐mastitis cows in study districts (*n* = 78).

		Number (%) bacteria isolated in milk from different districts				
Bacteria spp.	Udder status	Handeni *n* = 25	Kilosa *n* = 25	Lushoto *n* = 10	Mvomero *n* = 18	Total (%)
*Staphylococcus aureus*	Non‐mastitis	1 (1.3)	6 (7.7)	3 (3.9)	1 (1.3)	11 (14.1)
	Subclinical	3 (3.9)	6 (7.7)	2 (2.6)	4 (5.1)	15 (19.2)
	Clinical	0 (0.0)	0 (0.0)	0 (0.0)	2 (2.6)	2 (2.5)
*Staphylococcus epidermidis*	Non‐mastitis	2 (2.6)	2 (2.6)	0 (0.0)	2 (2.6)	6 (7.6)
	Subclinical	2 (2.6)	5 (6.4)	5 (6.4)	2 (2.6)	14 (18.0)
	Clinical	2 (2.6)	1 (1.3)	0 (0.0)	4 (5.1)	7 (9.0)
*Streptococcus agalactiae*	Non‐mastitis	0 (0.0)	0 (0.0)	0 (0.0)	0 (0.0)	0 (0.0)
	Subclinical	0 (0.0)	1 (1.3)	1 (1.3)	1 (1.3)	3 (3.9)
	Clinical	1 (1.3)	1 (1.3)	3 (3.9)	2 (2.6)	7 (9.0)
*Escherichia coli*	Non‐mastitis	1 (1.3)	2 (2.6)	0 (0.0)	0 (0.0)	3 (3.9)
	Subclinical	2 (2.6)	3 (3.9)	0 (0.0)	2 (2.6)	7 (9.0)
	Clinical	0 (0.0)	0 (0.0)	0 (0.0)	0 (0.0)	0 (0.0)
*Listeria monocytogenes*	Non‐mastitis	1 (1.3)	1 (1.3)	1 (1.3)	1 (1.3)	4 (5.1)
	Subclinical	6 (7.7)	5 (6.4)	3 (3.9)	2 (2.6)	16 (20.5)
	Clinical	0 (0.0)	0 (0.0)	0 (0.0)	5 (6.4)	5 (6.4)
*Listeria ivanovii*	Non‐mastitis	1 (1.3)	3 (3.9)	0 (0.0)	0 (0.0)	4 (5.1)
	Subclinical	4 (5.1)	1 (1.3)	0 (0.0)	3 (3.9)	8 (10.3)
	Clinical	0 (0.0)	1 (1.3)	0 (0.0)	0 (0.0)	1 (1.3)
*Listeria innocua*	Non‐mastitis	2 (2.6)	4 (5.1)	1 (1.3)	2 (2.6)	9 (11.5)
	Subclinical	8 (10.3)	4 (5.1)	1 (1.3)	1 (1.3)	14 (18.0)
	Clinical	0 (0.0)	0 (0.0)	0 (0.0)	1 (1.3)	1 (1.3)

**TABLE 6:** Bacteria isolates distributed according to cow breeds in the districts (*n* = 78).

		Number (%) of samples in different districts				
Bacteria spp.	Breed	Handeni *n* = 25	Kilosa *n* = 25	Lushoto *n* = 10	Mvomero *n* = 18	Total (%)
*Staphylococcus aureus*	Local	0 (0.0)	1 (1.3)	3 (3.8)	1 (1.3)	5 (6.4)
	Dairy	2 (1.3)	8 (10.3)	6 (7.7)	7 (9.0)	23 (29.5)
*Staphylococcus epidermidis*	Local	4 (5.1)	6 (7.7)	0 (0.0)	1 (1.3)	11 (14.1)
	Dairy	2 (2.6)	6 (7.7)	5 (6.4)	3 (3.9)	16 (20.5)
*Streptococcus agalactiae*	Local	0 (0.0)	1 (1.3)	1 (1.3)	1 (1.3)	3 (3.9)
	Dairy	1 (1.3)	1 (1.3)	3 (3.9)	2 (2.6)	7 (9.0)
*Escherichia coli*	Local	2 (2.6)	5 (6.4)	0 (0.0)	1 (1.3)	8 (10.3)
	Dairy	1 (1.3)	0 (0.0)	0 (0.0)	1 (1.3)	2 (2.6)
*Listeria monocytogenes*	Local	6 (7.7)	6 (7.7)	0 (0.0)	2 (2.6)	14 (17.9)
	Dairy	1 (1.3)	0 (0.0)	4 (5.1)	6 (7.7)	11 (14.1)
*Listeria ivanovii*	Local	3 (3.9)	5 (6.4)	0 (0.0)	0 (0.0)	8 (10.3)
	Dairy	2 (2.6)	0 (0.0)	0 (0.0)	3 (3.9)	5 (6.4)
*Listeria innocua*	Local	10 (12.8)	8 (10.3)	0 (0.0)	0 (0.0)	18 (23.1)
	Dairy	0 (0.0)	0 (0.0)	2 (2.6)	4 (5.1)	6 (7.7)

**FIGURE 2:**
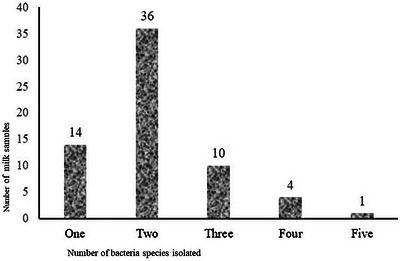
Number of bacterial species isolated from milk samples.

A total of 137 bacterial isolates detected were categorised as non‐mastitis were 37 (27.0%), subclinical 77 (56.2%) and clinical 23 (16.8%). The milk samples from TSHZ and dairy cattle that had bacterial infection were 25 (89.2) and 39 (78%), respectively. Of the 137 isolates of bacteria observed during the current study, 67 (48.9%) were from TSHZ, while 70 (51.1%) were from dairy cattle. The seven species of bacteria identified were *S. aureus* (20.4%), *S. epidermidis* (19.7%), *L. monocytogenes* (18.2%), *L. innocua* (17.5%), *L. ivanovii* (9.5%), *Str. agalactiae* (7.2%), and *E. coli* (7.3%). Most of the milk samples (78.5%) containing bacterial isolates exhibited mixed infections, with almost 70.6% of these samples showing the presence of two species (Figure 2). Up to 56.9% of milk samples from TSHZ contained mixed bacteria, whereas only 43.1% of samples from dairy cattle showed the same (Figure 2).

High infection rates by *S. aureus*, *S. epidermidis* and *Str. agalactiae* were observed in dairy cattle, while in TSHZ cattle, they were commonly infected by *L. innocua*, *L. monocytogenes* and *S. epidermidis*. Specifically, there were more isolates in Kilosa (33.6%; *n* = 47), while Lushoto had the lowest (17.5%; *n* = 24). Furthermore, Lushoto and Mvomero had more isolates of *S. aureus*, *S. epidermidis* and *L. monocytogenes* in dairy cows, while *S. epidermidis*, *S. aureus*, *L. monocytogenes* and *L. innocua* were common in TSHZ from Handeni and Kilosa.

### 4.5 Antimicrobial Susceptibility Test of Isolated Milk‐Borne Bacteria

A total of 137 bacterial isolates from milk culture‐positive samples (*S. aureus* = 28; *S. epidermidis* = 27, *Str. agalactiae* = 10, *E. coli* = 10, *L. monocytogenes* = 25, *L. ivanovii* = 13 and *L. innocua* = 24) were subjected to antimicrobial susceptibility tests, and the results are shown in Table 7. It was observed that more than 70% of the bacterial isolates were resistant to tetracycline, Nalidixic acid (NA) and amoxycillin, especially the Gram‐positive bacteria *S. aureus*, *S. epidermidis* and *Str. agalactiae*. Furthermore, *S. aureus*, *S. epidermidis*, *E. coli* and *L. monocytogenes* showed the highest resistance rates against Amoxycillin/Clavulanic acid (AMC), ampicillin and amoxycillin. In addition, most bacteria were sensitive to ciprofloxacin and gentamycin, especially *S. epidermidis*, *E. coli* and *Listeria* spp.

**TABLE 7:** Resistance patterns of isolates to antibiotics (*n* = 78).

	Number (%) of resistant bacteria						
	Antimicrobials tested						
Bacteria spp.	TET	CIP	GEN	NA	AMC	AMP	AML
*Staphylococcus aureus* (*n* = 28)	26 (92.9)	9 (32.1)	6 (21.4)	22 (78.6)	23 (82.1)	24 (85.7)	26 (92.9)
*Staphylococcus epidermidis* (*n* = 27)	25 (92.6)	5 (18.5)	15 (60.0)	25 (92.6)	18 (72.0)	20 (74.1)	25 (92.6)
*Streptococcus agalactiae* (*n* = 10)	9 (90.0)	4 (40.0)	2 (20.0)	6 (60.0)	2 (20.0)	4 (40.0)	8 (80.0)
*Escherichia coli* (*n* = 10)	7 (70.0)	2 (20.0)	2 (20.0)	5 (50.0)	3 (30.0)	8 (80.0)	8 (80.0)
*Listeria monocytogenes* (*n* = 25)	8 (32.0)	6 (24.0)	5 (20.0)	22 (88.0)	16 (64.0)	18 (72.0)	19 (76.0)
*Listeria ivanovii* (*n* = 13)	6 (46.2)	0 (0.0)	2 (15.4)	1 (7.7)	3 (23.1)	2 (15.4)	4 (30.8)
*Listeria innocua* (*n* = 24)	16 (66.7)	2 (8.3)	6 (25.4)	21 (87.5)	5 (20.8)	4 (16.7)	9 (37.5)

Abbreviations: AMC, amoxycillin/clavulanic acid; AML, amoxycillin; AMP, ampicillin; CIP, ciprofloxacin; GEN, gentamycin; NA, nalidixic acid; TET, tetracycline.

## 5 Discussion

This study assessed mastitis prevalence in lactating cows and the microbial quality of milk. The findings revealed a high prevalence of mastitis, particularly subclinical cases, which pose significant economic losses for smallholder farmers through reduced milk yield and quality. Studies show that economic losses due to subclinical mastitis range between $577 and $3239 per farm and $110 to $400 per cow (Mdegela et al. 2009; Ashraf and Imran 2020). Therefore, there is an urgent need to control this problem. Most marketed milk is contaminated with diverse bacterial species, including pathogens with the potential to cause mastitis in cattle and milk‐borne diseases in humans. Nevertheless, the majority of milk samples had mixed bacterial infections that exhibited resistance to tetracycline, Nalidixic acid (NA) and penicillins, underscoring urgent animal and public health concerns. Pasteurisation and improved control measures are recommended to mitigate these risks.

The current study has established a high prevalence (75.6%) of mastitis in lactating cows in the study area. These findings could be related to animal husbandry and milk handling practices that are undertaken in the districts. There are several practices at the farm level, such as animal house floors, not washing hands and udder and teats before milking and many unhygienic practices (Kanyeka 2014). Furthermore, milking sick animals and those with udder problems before milking non‐mastitis cows further facilitates mastitis bacteria transmission (Kanyeka 2014; Gwandu et al. 2018). Water used for cleanliness in washing hands and milk equipment is not clean and may be a potential source of infection, as previously reported (Swai and Schoonman 2011; Kanyeka 2014). In the study districts, smallholder livestock farmers practised hand milking in cattle houses that were consistently filled with cow dung, which may explain the high prevalence of mastitis among cows. To control mastitis in cows in the study areas, farmers are advised to observe good livestock husbandry and hygienic practices in the animal houses, use proper hand milking procedures, apply pre‐ and post‐milking teat dips, and perform routine screenings using simple equipment such as strip cups and CMT.

It was further found that dairy cattle were more affected by mastitis at a rate of 82.1% compared to local cattle (TSHZ) at 72%, but this difference was not statistically significant (*p* = 0.3168). Although the results showed no statistically significant difference in mastitis prevalence, previous studies in Tanzania have reported that dairy cattle are more frequently affected than local breeds (Karimuribo et al. 2006; Mbeho 2012). Elsewhere, a study by Abdel‐Rady and Sayed (2009) in Egypt found the Friesian breed to be more affected by mastitis (20.4%) than the native breed (16.7%). These discrepancies with the present study may be attributable to differences in sample size, animal management practices, study design and the diagnostic methods employed. Generally, improved breeds of dairy cattle are prone to mastitis, possibly due to high milk production, intense milking, poor hygiene, compromised immunity during lactation and nutritional stress (Rahularaj et al. 2019; Mramba and Mohamed 2024). In contrast, local cattle are tolerant of harsh environments and diseases (Laswai and Nandonde 2014).

Subclinical mastitis was more prevalent (62.8%) than clinical cases, as previously reported (Abdel‐Rady and Sayed 2009). The subclinical form of mastitis is considered 15–40 times more prevalent than the clinical form and accounts for greater losses in terms of milk production, and it represents a reservoir of infectious organisms (Mdegela et al. 2009; Mbeho 2012). Subclinical mastitis is associated with different factors, including breeding distribution, different seasons and different age groups (Abdel‐Rady and Sayed 2009). The current study was conducted during the dry season, which may support the high incidence of mastitis in milking cows because of environmental exposure (Mbeho 2012; Rahularaj et al. 2019; Mramba and Mohamed 2024).

For the first time, *Listeria* species have been isolated in Tanzania at a rate of 18.2% and are among the common species encountered in this study. Other *Listeria* species isolates were *L. ivanovii* and *L. innocua*. The high prevalence of *Listeria* spp. in the study area may be attributed to unhygienic practices, contamination of the udder, the surrounding environment and milking equipment (Kanyeka 2014; Castro et al. 2018). Interestingly, *S. aureus* accounted for most of the isolated bacteria in milk, especially from dairy cows. However, other common bacteria were *S. epidermidis*, *L. innocua*, *L. ivanovii*, *Str. agalactiae* and *E. coli* are the main microbial milk contaminants. Bacteria are the primary cause of contagious mastitis, occurring in both clinical and subclinical forms in milking cows, and they also represent the main microbial contaminants of milk (Almaw et al. 2008). Other studies observed similar results in Tanzania and elsewhere, showing the commonality of mastitis‐causing agents (Abdel‐Rady and Sayed 2009; Mbeho 2012; Hosseinzadeh and Saei 2014; Kilyenyi et al. 2021).

Consumption of milk contaminated with *S. aureus* can be a health hazard because approximately 10% of mastitis staphylococci are known to be producers of enterotoxins, which are heat‐stable toxins (Vasil et al. 2016; Regecová et al. 2022). Some studies have associated *S. aureus* with gastroenteritis through these enterotoxins (Ngasala et al. 2015; Regecová et al. 2022). Since *S. aureus* is a contagious and common coloniser of teat ends and teat canals, the use of dry cows and post‐milking teat disinfectants can be of great value in the control of mastitis (Ozbey et al. 2022; Vargová et al. 2023). Similarly, *E. coli* was also isolated from milk samples, suggesting faecal contamination and causing environmental mastitis (Ngasala et al. 2015; Regecová et al. 2022; Ozbey et al. 2022). Some strains are verocytotoxigenic, such as the enterohaemorrhagic type of *E. coli* O157:H7, and cause haemorrhagic colitis (Regecová et al. 2022; Xu et al. 2025). Indeed, a study by Schoder et al. (2013) reported the occurrence of *E. coli* O157:H7 in raw milk from traditional cattle farms in Tanzania. Similarly, Lupindu et al. (2014) isolated a highly pathogenic *E. coli* O157:H7 in cattle manure in Morogoro, which further gives danger when *E. coli* is isolated in milk.

The current study isolated bacteria that were resistant to many antimicrobials tested, particularly against tetracycline, Nalidixic acid (NA) and amoxycillin implying that there is indiscriminate use of such antibiotics. In Tanzania, antimicrobials are used for the treatment of ill health regardless of diagnosis, which always leads to misuse or indiscriminate use (Katakweba et al. 2012; Sangeda et al. 2021; Mdegela et al. 2021; Mashauri et al. 2025). However, misuse of antimicrobials is driven by high disease burdens, limited availability of diagnostic, limited extension services, economic pressures to maintain herd productivity, easy access to over‐the‐counter antimicrobials, arbitrary drug combinations and the acquisition of mobile genetic characteristics (Katakweba et al. 2012; Kimera et al. 2020; Sangeda et al. 2021; Mdegela et al. 2021; Mashauri et al. 2025). Indeed, mastitis is among the common health problems in smallholder dairy farms, and its management relies on clinical judgment, with limited microbiological diagnostic support, antimicrobial susceptibility tests and parenteral antimicrobials used empirically as first‐line therapy (Mdegela et al. 2021; Muloi et al. 2025; Kimambo et al. 2026). Improving preventive measures and antimicrobial stewardship can reduce the risk of AMR.

### 5.1 Study Limitations

This cross‐sectional study was conducted during the dry season and involved clinical examination of cows for mastitis, as well as collection of milk samples from smallholder cattle farmers for bacterial culture and antimicrobial sensitivity testing, in four of Tanzania's 184 district councils. Therefore, although the results represent the general picture of the real situation of mastitis and AMR in dairy and local breed cows in Tanzania, the small sample size of 78 cows provides baseline information that warrants a larger longitudinal study. In addition, animal biodata, including age, stage of lactation, parity and drying practices with or without antibiotic use, were not recorded during data collection, limiting the ability to correlate these factors with mastitis occurrence in lactating cows.

### 5.2 Conclusions and Recommendations

This study reports that the preliminary prevalence of clinical and subclinical mastitis was high in the study districts and was caused by seven bacterial species, dominated by *S. aureus*, *S. epidermidis* and *L. monocytogenes*, which showed high AMR rates. The reported prevalence of mastitis, the bacterial isolates and their AMR were constrained by the small sample size, which involved only smallholder cattle farmers, and by the fact that the study was conducted exclusively during the dry season in just four of Tanzania's 184 districts. Nevertheless, urgent intervention measures are required to reduce farmers' losses due to mastitis and to safeguard public health against milk‐borne diseases and AMR. The available legislation strictly limits uncontrolled access and use of veterinary drugs, but they are not enforced.

## Author Contributions

**G. Msalya**: conceptualisation, funding acquisition, methodology, validation, writing – review and editing, formal analysis, project administration, supervision, resources. **C. Faustine Ndyamukama**: conceptualisation, investigation, writing – original draft, methodology, formal analysis. **Hezron Emmanuel Nonga**: conceptualisation, funding acquisition, methodology, visualisation, writing – review and editing, software, project administration, data curation, supervision, resources.

## Funding

German Society for International Cooperation (GIZ) and the Consultative Group on International Agricultural Research (CGIAR) for funding my research work through the Safe Food, Fair Food (SFFF II).

## Ethics Statement

The research was conducted according to the guidelines provided by the code of ethics of Sokoine University of Agriculture. A research permit was obtained from Sokoine University of Agriculture on behalf of the Commission of Science and Technology (COSTECH). Permissions to conduct research in Kilosa, Mvomero, Lushoto and Handeni districts were provided by the District Executive Directors, and every time, the District Veterinary Officers or the District Livestock Officers accompanied the researchers. Verbal permits were sought from Ward Executive Officers (WEOs) and Village Executive Officers (VEOs). Verbal consent was also requested from each participating smallholder farmer. Heads of household signed the consent form, and participation in the study was voluntary. All the data that were collected from the respondents and the laboratory results obtained after milk sample analysis were kept under the custody of the researcher as confidential, and the study participants were anonymised.

## Conflicts of Interest

The authors declare no conflicts of interest.

## Data Availability

The data generated through this study are contained in the manuscript. Additional data sets are available upon request from the authors.
